# Soy Protein Isolate-Stachyose Emulsion Gel for the Delivery of Vitamin D_3_: Effect on the Humoral Immune Response in Dairy Goats Under Heat Stress

**DOI:** 10.3390/ani15172588

**Published:** 2025-09-03

**Authors:** Adela Mora-Gutierrez, Maryuri T. Núñez de González, Rahmat Attaie, Yoonsung Jung

**Affiliations:** 1 Cooperative Agricultural Research Center, Prairie View A&M University, Prairie View, TX 77446, USA; mtnunez@pvamu.edu (M.T.N.d.G.); rattaie@pvamu.edu (R.A.); 2 Statistical Consulting Center, Department of Statistics, Texas A&M University, College Station, TX 77843, USA; yojung@exchange.tamu.edu

**Keywords:** vitamin D_3_, delivery system, nuclear magnetic resonance, hydration, prebiotics, heat stress, immune response, dairy goats

## Abstract

Small ruminant production is a significant sector of agricultural industry in Texas, USA. Heat stress has a negative effect on productivity and animal health. Cholecalciferol, a form of vitamin D_3_, may enhance the function of immune cells and help ensure healthy immune function in farm animals exposed to heat stress. Practical applications of vitamin D_3_ against infectious diseases can benefit from the protective effects of a delivery system comprised of soy protein isolate and stachyose in emulsion gel. The prebiotic oligosaccharide stachyose has shown to have a great potential as a substrate for beneficial intestinal bacteria, which are thought to modulate the immune system. Cellular and humoral immunity are both impaired in dairy animals under heat stress. The delivery of vitamin D_3_ embedded within the soy protein isolate-stachyose emulsion gel resulted in a marked increase in 25-hydroxyvitamin D_3_ [25-(OH)-D_3_] concentration in blood serum. Chicken egg albumin (OVA)-immunized goats produced low anti-OVA immunoglobulin G (IgG) responses. In contrast, OVA-immunized goats fed vitamin D_3_ within the soy protein isolate-stachyose emulsion gel diet strongly stimulated antibody production. These results show that anti-OVA IgG responses can be modulated in dairy goats using vitamin D_3_, particularly if this vitamin is delivered in the form of emulsion gel. The results seem to depend on the highly hydrated gel matrix of soy protein isolate-stachyose at the low pH of the stomach as monitored by oxygen-17 (^17^O) and proton (^1^H) nuclear magnetic resonance (NMR). In addition, the prebiotic nature of stachyose may boost beneficial gut bacteria, most notably for immune health and reducing the risk of infectious diseases.

## Simple Summary

Heat stress is one of the limiting factors in sustainable production in small ruminants, especially in hot and humid climates. Humoral immunity, a critical component of the adaptive immune system, relies heavily on antibodies, also known as immunoglobulins. Immunoglobulin G (IgG) is the most abundant antibody found in blood and extracellular fluids of dairy animals. In this study, we found that vitamin D_3_ administration can restore IgG responses to specific antigens, i.e., chicken egg albumin (OVA). Vitamin D_3_ embedded within soy protein isolate-stachyose emulsion gel strongly stimulated anti-Ova IgG production in dairy goats under heat stress. These findings provide valuable insights for dairy goat producers to reduce the incidence of bacterial sub-clinical mastitis by reversing the immunosuppression associated with heat stress.

## 1. Introduction

Deficiency of cholecalciferol (vitamin D_3_) has been shown to increase susceptibility to infections, whereas vitamin D_3_ administration has caused a drastic decrease in the incidence of infections [1]. Cholecalciferol hydroxylation in the liver produces 25-hydroxy cholecalciferol [25-OH-D_3_], which may be transformed further in the kidney to other derivatives of vitamin D_3_. Serum concentrations of 25-hydroxyvitamin D_3_ indicate whether vitamin D_3_ status is adequate. Currently, the animal feed industry is interested in the potential utilization of 25-hydroxyvitamin D_3_ as an alternative to cholecalciferol [2]. High serum concentrations of 25-hydroxyvitamin D_3_ positively affect animal performance and their health status [3]. Immunoglobulins, also known as antibodies, are produced by white blood cells and make important contributions to immunity. Among these immunoglobulins, IgA has a primary defense role against pathogens and activates mucosal immunity [4]. Dairy herds with low serum IgA levels are readily exposed to diseases, such as mastitis [5]. Complex coacervates of sulfur lactoferrin and sodium alginate crosslinked with microbial transglutaminase in the spray-dried form have been shown to be a good vitamin D_3_ supplement in dairy goats’ feed. They increased the immune response of late-lactating dairy goats [6].

Defatted soy flakes are used to extract soy protein isolates with a minimum heat process. Soy protein isolates usually contain 90% protein, and since it is carbohydrate- and fat-free, they do not retain a “beany” flavor. Soy protein isolate is usually used in many infant formulas and medical nutritional products due to its bland flavor and high-quality protein [7,8]. Moreover, stachyose is a prebiotic oligosaccharide with biological activities such as regulating intestinal microflora and alleviating inflammatory response and oxidative stress when used in combination with *Lactobacillus rhamnosus* GG [9]. It is generally accepted that oligosaccharide prebiotics, such as inulin and oligofructose, modulate immunological processes at the level of the gut-associated lymphoid tissue (GALT) [10]. The intestinal bacteria are present mainly in the large intestine. Therefore, in order to increase the quantity of beneficial intestinal microflora by the ingestion of prebiotic oligosaccharides, these compounds must escape the digestion and the absorption processes of the small intestine and reach the large intestine. Similar to the other prebiotics, stachyose has these properties. The intestinal bacteria metabolize prebiotic oligosaccharides readily and produce large amounts of short-chain fatty acids. As a result, the pH in the lumen of the large intestine decreases to an acidic pH value (1.5 to 3.5). At the same time, the total number of intestinal microbes increases and enhances the fecal volume. The beneficial bacteria, such as *Bifidobacterium* spp. and *Lactobacillus* spp., are resistant to the acidic environment, whereas the harmful bacteria, such as *Clostridium* spp., are sensitive to the acidic conditions [11]. The increase in the growth of *Bifidobacterium* spp. is accompanied by the production of nitrogen derivatives, such as ammonia, indole, phenol, and skatole, and by the elimination of carcinogenic substances during the fermentation [12].

The underlying physicochemical properties of soy protein isolate-stachyose emulsion gel have been outlined elsewhere [13]. Enteral (orally consumed) vitamin D_3_ embedded within the soy protein isolate-stachyose emulsion gel to rats affected the 25-(OH)-D_3_ plasma levels (the main circulating form of vitamin D_3_) with a larger area under concentration-time curve at 0 and 24 h (AUC_0–24h_) and higher maximum concentration (C_max_) [13]. The soy protein isolate-stachyose emulsion gel should, in theory, serve as a substrate to *Bifidobacterium* spp. and other useful bacteria, facilitating their growth in the large intestine. The supplementation of vitamin D_3_ embedded within the soy protein isolate-stachyose emulsion gel may contribute to the protection of dairy goats against mastitis, since dietary vitamin D_3_ is associated with immunomodulatory properties [14]. There is also sufficient evidence to suggest that prebiotic-formulated diets have beneficial effects for gut microbiota, metabolic activity, stool consistency and frequency, and the development of some immune markers [15].

Heat stress constitutes a significant cost for the dairy industry [16]. Studies in animal models have demonstrated that heat stress decreases immune function and resistance to infection [17,18,19]. In this context, heat stress impacts both cell-mediated (T-lymphocytes) and humoral (B-lymphocytes) responses. Vitamin D_3_ can modulate these responses, thereby maintaining immune homeostasis during heat stress [20]. This study aims to evaluate the effects of soy protein isolate-stachyose emulsion gel as a vehicle for the delivery of vitamin D_3_ in healthy dairy goats under heat stress. We hypothesized that the inclusion of a low dose of vitamin D_3_ embedded within the soy protein isolate-stachyose emulsion gel in the diet of healthy dairy goats can modulate the humoral immune response under heat stress.

## 2. Materials and Methods

### 2.1. Materials

Cholecalciferol (vitamin D_3_), deuterium oxide (99.8%; D_2_O), dimethyl sulfoxide (DMSO), monobasic potassium phosphate, calcium chloride, sodium chloride, PIPES buffer, stachyose, microbial transglutaminase, lipase, bile salt, and ovalbumin were purchased from Sigma-Aldrich (St. Louis, MO, USA). Soy protein isolate obtained using alkali-solution and acid-solution method was a gift of Cargill (Wayzata, MN, USA). The Stepan Company (Northfield, IL, USA) donated the medium-chain triglycerides (MCT) oil that had the following specifications: Neobee M-5, ≥66% C8:0, and ≥32% C10:0. All solvents, chemicals, and reagents that were used for the extraction and analysis of samples were either HPLC-grade or analytical grade and purchased from Sigma-Aldrich. Deionized water was prepared by passing distilled water over a mixed-bed cation-anion exchanger and used throughout this study.

### 2.2. Preparation of Emulsion Gel

Soy protein isolate (final concentration 10%) and stachyose (final concentration 4%) were dispersed in deionized water, respectively. The solution was continuously stirred for 3 h at room temperature. The final pH of the emulsion was adjusted to 6.0 using 0.5 M HCl. Next, 15% (*v*/*v*) MCT oil with 0.2% (*w*/*w*) vitamin D_3_ (cholecalciferol) was added and mixed with a hand-held homogenizer (Biospec Products Inc., Bartlesville, OK, USA) at low speed for 2 min at 20 °C. The coarse gel emulsion was homogenized twice at 20 MPa (12,000 psi) through a high-pressure TC5 homogenizer (Stansted Fluid Power, Harlow, UK). Calcium (10 mM) and microbial transglutaminase (30 U/g of soy protein isolate) were added to the gelled emulsion and then incubated in a water bath at 50 °C for 2 h. Subsequently, the microbial transglutaminase was inactivated by holding the gelled emulsion samples at 85 °C for 15 min and then cooled in an ice bath immediately. The emulsion gel was molded into small disks and sealed in alumina blister trays. The blister trays were stored under nitrogen atmosphere at 4 °C.

### 2.3. Fourier-Transform Infrared (FTIR)

Fourier-transform infrared (FTIR) spectroscopy was used to characterize the structure of the soy protein isolate-stachyose delivery system loaded with vitamin D_3_ dispersed in DMSO or MCT. The emulsion gel was cut into thin slices (1 mm thick) and then freeze-dried (LABCONCO, Kansas City, MO, USA). The freeze-dried emulsion gel (300 mg) was dissolved in 2.5% *w*/*w* aqueous solutions at pH 7.0 in PIPES buffer. The FTIR spectra were recorded between 500 and 4000 cm^−1^ with 35 scans per sample, using a FTIR spectrometer (Nicolet Model 740, Madison, WI, USA) equipped with a Nicolet 660 data system. Samples were introduced in a temperature-controlled demountable cell (Sperac Inc., Smyna, CA, USA). The cell had CaF_2_ windows that were separated by a 6 μm Mylar spacer. Resolution was 1 cm^−1^, with 1024 double-sided interferograms collected for each spectrum.

### 2.4. X-Ray Diffraction

X-ray diffraction (XRD) patterns were recorded to assess the crystalline nature of vitamin D_3_ and the soy protein isolate-stachyose emulsion gel loaded with vitamin D_3_ dispersed in DMSO using a Nicolet 740 FTIR spectrometer (Madison, WI, USA) equipped with a Nicolet 660 data system. All samples were vacuum-dried for analysis.

### 2.5. In-Vitro Dissolution Testing of the Emulsion Gel

Gelled emulsion (1 g) was cut into small pieces (mean particle diameter: 3–4 mm) to mimic the fracture during chewing. The fractured gel emulsion was added to 150 mL pre-warmed simulated gastric fluid (SGF) and incubated at 37 °C and 50 rpm for 24 h. The SGF, pH 1.2, without enzymatic activity, was prepared accordingly [21]. After the incubation period, the concentration of vitamin D_3_ was determined by HPLC-UV [22].

An aliquot (20 mL) of SGF was adjusted to pH 7.0 by adding 0.3 mol NaOH and then adding 10 mL of simulated intestinal fluid SIF (2 mg/mL lipase, 20 mg/mL bile salt, and 2 mmol/L CaCl_2_). The prepared SIF was preheated at 37 °C for 5 min, and the mixtures were continuously stirred at 37 °C and shaken at 200 rpm/min for 24 h, and we adjusted the pH of the mixture back to 7.0 by adding 0.3 mol NaOH.

### 2.6. Light Scattering In-Vitro Measurements of Oil Droplet Size

Droplet size measurements were performed at different SGF incubation times at 21 °C with a SALD-2101 laser diffraction particle analyzer (Shimadzu, Columbia, MD, USA), to demonstrate that emulsions induced by dissolution of the gelled emulsion were stable at artificial gastric conditions. Dissolved matrix was applied to the instrument (from *in*-*vitro* dissolution) in triplicate at each SGF incubation time.

### 2.7. Zeta Potential In-Vitro Measurements of Oil Droplets

The charge of the oil droplets was performed at different SFG incubation times at 21 °C with a Zetasizer Nano ZS (Malvern Instruments, Worcestershire, UK) to demonstrate that the emulsions induced by dissolution of the gelled emulsion were stable at artificial gastric conditions. Dissolved matrix was applied to the instrument (from *in*-*vitro* dissolution) was applied to the instrument in triplicate at each incubation time (0 h to 24 h). Samples were diluted 100 times in 5 mM phosphate buffer at pH 7.6.

### 2.8. ^17^O NMR Measurements

The hydration properties of the soy protein isolate alone and in combination with stachyose were determined by ^17^O NMR according to the method described by Mora-Gutierrez et al. [23]. To study their interactions, soy protein isolate at a fixed concentration of 2% *w*/*w* and stachyose at different concentrations (0, 0.1, 0.2, 0.4, and 0.8%, *w*/*v*) were mixed in deuterated phosphate-buffered saline (PBS) at pH/pD values of 2.4, 6.5 and 7.6 at 21 °C in the presence of 0.4 M NaCl under constant stirring. Conversion to pD values was made according to the relation pD = pH + 0.4, where pH is the pH-meter reading for a solution in D_2_O with the electrode calibrated in standard H_2_O buffers [24]. The ionic strength of 0.4 M simulates the gastrointestinal (GI) tract [25]. Samples were transferred to 10-mm high-resolution NMR tubes (Sigma-Aldrich). ^17^O NMR experiments were performed at 21.1 Tesla using a Bruker Avance II 300 MHz spectrometer (Bruker Corporation, Billerica, MA, USA). A recycle delay of 0.5 s was used. The number of scans required for a good signal-to-noise ratio in the ^17^O NMR spectra of the samples was about 2000. A spinning frequency of 23 kHz was utilized. Single radiofrequency pulses of 27 μs pulsewidth (90° flip angle) in the presence of broadband decoupling at 300 MHZ were used.

### 2.9. ^1^H NMR Measurements

^1^H NMR relaxation times (T_1_ and T_2_) were performed at 20 °C on a Bruker Avance II 300 MHz spectrometer (Bruker Corporation) according to the method described by Mora-Gutierrez and Baianu [26]. T_1_ was derived from a series of spectra collected using a standard inversion-recovery pulse sequence (180-τ-90-delay), in which the variable delay, τ, was varied from 100 ms to 40 s. T_2_ was measured using the standard Carr–Purcell–Meiboom–Gill (CPMG) pulse sequence, (90-τ-1800*_n_*-delay), in which *n* was varied from 2 to 256 for the 2.5% stachyose gel, decreasing to a range of 2 to 128 for the 12.5% stachyose gel (ι = 1 ms for all measurements). Relaxation rates, R_1_ and R_2_, were calculated as the reciprocal value of relaxation times, T_1_ and T_2_, respectively. Relaxation times (T_1_ and T_2_) were derived from fitting the series of integrated peaks to standard exponential functions using Origin 7 (OriginLab Corporation, Northampton, MA, USA). Six measurements were performed on each sample to determine an average value and associated uncertainty for T_1_ and T_2_. All quoted uncertainties are repeatability standard deviations multiplied by a factor of 2.36 to give the 95% confidence interval.

The hydrogen spectra of the soy protein isolate-stachyose gelled emulsion samples at concentrations of 2.5% *w*/*w* and 0.3% *w*/*w*, respectively, and pH/pD 2.4 and 7.6 were determined by ^1^H NMR using a 400 MHz Bruker instrument (Bruker Corporation). The soy protein isolate-stachyose gelled emulsion samples were cut into 1 cm^3^ and placed in the sample tube. The sample tube was placed in the radiofrequency (RF) coil. A pulse width of 30 μs (77 °C mutation angle) and a spectral width of 6 kHz were used; the acquisition time and the recycle time were 1.4 and 3.4 s, respectively. Data were stored in a 16 K memory block. Approx. 30 min were allowed for each sample to reach thermal equilibrium in the magnet before data acquisition.

### 2.10. Determination of Embedding Rate

The aqueous suspension of vitamin D_3_ emulsion gel prepared in Section 2.2 was mixed with anhydrous ethanol and centrifuged at low speed for 5 min at 20 °C to separate free vitamin D_3_. The supernatant after centrifugation was collected and diluted with anhydrous ethanol. The quantitative determination of vitamin D_3_ was performed using HPLC-UV [22]. The embedding rate (ER) was calculated as follows:

(1) $$\mathrm{ER}\;(\%)=(\left(1\;-\;\mathrm{free}\;\mathrm{vitamin}\;D_{3}\;\mathrm{content}\right)/\mathrm{total}\;\mathrm{vitamin}\;D_{3}\;\mathrm{content})\;\times \;100$$

The stability of vitamin D_3_-loaded gel emulsions was evaluated during storage at 4 °C under nitrogen gas for 6 months. Vitamin D_3_ content was measured at the 1st, 5th, 10th, 15th, 30th, 60th, 90th, and 180th days. The content of vitamin D_3_ was determined by HPLC-UV [22].

### 2.11. Differential Scanning Calorimetry (DSC)

The freeze-dried samples were rehydrated to 30 g dry matter/100 g. Samples of 30–40 mg were weighed into pressure pans (Perkin Elmer Inc., Norwalk, CT, USA). The measurements were carried out on the 2910 DSC (TA Instruments, New Castle, DE, USA) that had been calibrated with indium and an empty pan as reference. The samples were heated at a rate of 10 °C/min from 4 to 160 °C with a nitrogen flush (40 mL/min). After cooling the sample to 4 °C with an average cooling rate of ~20 °C/min, a second run was performed. Phase transition characteristics were evaluated using the TA Instruments software program (TRIOS version 5.1). 

### 2.12. Animals, Diets, Facilities, and Experimental Design

In a 56-day feeding trial, eighteen Alpine dairy goats (mean age 2.5 years, mean BW 114.2 kg) were selected from the milking herd of the International Goat Research Center (IGRC) at Prairie View A&M University, Prairie View, TX, USA. All experimental procedures with the goats were in strict compliance with the current guidelines and legal requirements established in the United States for the proper use and care of animals and approved by the Institutional Animal Care and Use Committee at Prairie View A&M University (Protocol # 2023-051). Six goats per experimental group (*n* = 18) were used, and at least 4 replicates were used for this experiment. Our sample size also follows the recommendations of the Institutional Animal Care and Use Committee at Prairie View A&M University for discovery experiments, where at least 4 biological replicates are needed. Udders were confirmed to be healthy by culturing udder half milk onto 5% blood agar plates and assessing over 48 h.

All goats were kept individually housed indoors at the IGRC, where the temperature and humidity were approximately 39 °C and 46%. Air temperature and relative humidity were monitored daily [12 h day (from 0800 to 2000), and 12 h night (from 2000 to 0800)] using a relative humidity/temperature meter (Fisher Scientific, Waltham, MA, USA). Temperature-humidity index (THI) was 84. Rectal temperatures were recorded daily at 0800, 1200, and 1700 (Table 1). The rectal temperature was measured with a digital clinical thermometer (Thermo Fisher Scientific, Waltham, MA, USA). The selected dairy goats were randomly assigned to three experimental groups: control (*n* = 6), vitamin D_3_ supplemented (0.35 mg vitamin D_3_/day) dispersed in MCT oil (*n* = 6), and vitamin D_3_ supplemented (0.35 mg vitamin D_3_/day) embedded in the emulsion gel (*n* = 6). After the initial 14-day adaptation period, the experiment continued for 56 days. All goats were at midlactation (162 days in milking; 864 mL milk/day) and were randomly assigned to an individual feeding gate on the day of experiment initiation.

The two treatments of dairy goats that were given the two delivery forms of vitamin D_3_ consisted of 0.35 mg of vitamin D_3_/day in addition to the base ration that provided 0.01 mg of vitamin D_3_/day. A top-dress supplement for the treatment with vitamin D_3_ was prepared by mixing 0.6 g of the vitamin D_3_ emulsion gel with cottonseed meal to provide 0.35 mg of vitamin D_3_ in 100 g of total mixture. A top-dress supplement for the treatment with vitamin D_3_ dispersed in MCT oil was prepared by combining vitamin D_3_ in MCT oil (0.35 mg vitamin D_3_/5 mL of MCT oil), homogenized for adequate dispersal, and mixed with cottonseed meal to provide 0.35 mg of vitamin D_3_ in 100 g of total mixture. The two top-dress supplements were not mixed into the ration and were consumed readily by all goats upon delivery. A commercially available concentrate was used as the basal diet, containing the following ingredients: wheat middling, corn, cottonseed meal, soybean hull, soya meal, ground limestone, soybean oil, sodium bicarbonate, salt mixing, monocalcium phosphate, vitamins, magnesium oxide, trace mineral premix, and selenium yeast. The chemical composition of the basal diet used in this study is shown in Table 2. The control and experimental animals were given 2.07 kg of basal diet twice a day, in the morning and in the evening, and the leftovers were measured. The diets supplemented with the two deliverable forms of vitamin D_3_ were only added to the morning feeding as a top-dress. Hay and water were available to animals *ad libitum*.

### 2.13. Sample Collection and Analysis of 25-(OH)-D_3_

Blood samples (5 mL) were collected by puncture of the jugular vein using siliconized needles (21 G × 1″) with a vacuum system. The blood samples taken on days 0 and 56 were transferred into 10 mL vacuum tubes (serum separator tube) (Becton Dickinson Vacutainer Systems, Franklin Lakes, NJ, USA) for 25-25-(OH)-D_3_ measurements. Tubes were centrifuged at 3500× *g* for 5 min in a refrigerated centrifuge (4 °C) for serum separation within 30 min of sample collection. Serum samples were transferred into microtubes using Pasteur pipettes. The samples were maintained at 2–8 °C and immediately analyzed, avoiding freeze-thaw cycles because they are detrimental to many serum components. Serum analyses were performed in triplicate. Blood serum 25-(OH)-D_3_ concentrations were quantified using a CDC-certified LC-MS method [28].

### 2.14. Establishment of Humoral Response to a Nominal Antigen Chicken Egg Albumin (OVA)

At the start of the experiment (0 day), 2 mg of chicken egg albumin (OVA) (Sigma-Aldrich) dissolved in 1 mL of sterile saline solution and 1 mL of Freund’s adjuvant (Sigma-Aldrich) were injected subcutaneously into both shoulders of each goat [29]. A subsequent injection of 2 mg OVA in saline without adjuvant was administered 15 days later. A single subcutaneous dose of 2 mg of OVA in sterile saline solution with and without adjuvant is safe for goats [29]. Swelling at the injection site was not observed. The administration of OVA was well tolerated by all dairy goats throughout the study period.

Blood was taken from the caudal vein of each goat at 0, 14, 28, 42, and 56 days of the experiment. Samples from each animal were collected into duplicate heparinized vacuum tubes (Becton Dickinson Vacutainer Systems) for each animal. Blood samples were centrifuged at 1200× *g* for 15 min at 25 °C to separate the plasma fraction. Plasma samples were collected, aliquoted in 6 replicates for each animal, and then stored at −80 °C to perform ELISAs to evaluate the anti-OVA IgG titers.

### 2.15. Anti-OVA Specific IgG by ELISA

The anti-OVA antibody titer in blood plasma samples was evaluated by an ELISA test [29] performed in 96-well U-bottomed microtiter plates. Wells were coated with 100 μL of antigen (10 mg of OVA/mL of phosphate-buffered saline (PBS) at 4 °C for 12 h, washed, and incubated with 1% skimmed milk (200 μL) at 37 °C for 1 h to reduce non-specific binding. After washing, the plasma (1:5000 dilution in PBS; 100 μL per well) was added and incubated at 37 °C. The extent of antibody binding was detected using a horseradish peroxidase-conjugated donkey anti-bovine IgG (Sigma-Aldrich) (1:20,000 dilution in PBS; 100 μL per well). Optical density was measured at a wavelength of 450 nm, and plasma samples were read against a standard curve obtained using scalar dilution of goat-specific IgG (ZeptoMetrix Corporation, Franklin, MA, USA). Data were expressed as mg of anti-OVA IgG/mL.

### 2.16. Statistical Analysis

Quantitative data for the combination of response and treatment variables are summarized with mean ± standard error. All tests were under 5% significance level. The data from hydration estimates of soy protein isolate with added stachyose and anti-OVA IgG concentrations in dairy goats fed with experimental diets under heat stress were analyzed by two-way ANOVA with Tukey test. A paired *t*-test under pre-post-test design was performed to compare the difference between before supplementation (day 0) and after supplementation (day 56) with animals assigned to different dietary groups. Experimental data were analyzed using SAS software (version 9.4, SAS Institute, Cary, NC, USA).

## 3. Results and Discussion

### 3.1. Storage Stability

A novel emulsion gel comprised of soy protein isolate-stachyose, as a vehicle for the delivery of vitamin D_3_, attained a high entrapment efficiency of 91.2%. The emulsion gel contained vitamin D_3_ at a concentration of 0.35 mg per gram (0.035%) as determined by HPLC-UV [22]. Formation of the emulsion gel matrix is a complex event generally understood to be affected by protein concentration, quantity, and state of water, ionic type and strength, heating/time temperature, pH, and interactions with other components such as the oligosaccharide stachyose. As to the storage stability of the vitamin D_3_ embedded in the emulsion gel, there was little change in concentration of about 0.1% after 3 months of storage at 4 °C. Six months later, the storage stability decreased by 1.4% at 4 °C, which was not significant. These results suggest that vitamin D_3_ embedded in the emulsion gel has good stability.

### 3.2. Thermal Stability

DSC measurements of vitamin D_3_-loaded gel emulsions suggest that the soy protein isolate-stachyose mixture enhances the thermal stability of the emulsion gels at 50 °C due to the addition of calcium and microbial transglutaminase as described in Section 2.2. The thermal denaturation temperature and helix content increase after sufficient transglutaminase cross-linking, and both have positive impact on the thermal stability of the emulsion gel.

### 3.3. FTIR Spectroscopy

**Figure 1:**
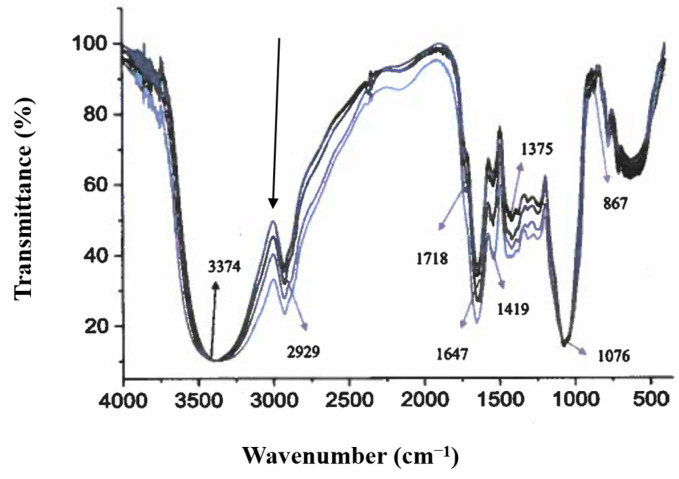
FTIR spectra from top to bottom as indicated by the large arrow of soy protein isolate, soy protein isolate-stachyose mixture, soy protein isolate-stachyose mixture loaded with vitamin D_3_ dispersed in DMSO, and soy protein isolate-stachyose mixture loaded with vitamin D_3_ dispersed in MCT.

The characteristic FTIR spectra of soy protein isolate, soy protein isolate-stachyose mixture, soy protein isolate-stachyose mixture loaded with vitamin D_3_ dispersed in DMSO, and soy protein isolate-stachyose mixture loaded with vitamin D_3_ dispersed in MCT are displayed in Figure 1. The soy protein isolate and soy protein isolate-stachyose mixtures loaded with vitamin D_3_ have similar IR absorption. The broad and intense absorption peak at approximately 3374 cm^−1^ is attributed to the -OH stretching vibration, whereas the weak band at approximately 2929 cm^−1^ has been assigned to the C-H stretching vibration of the alkyl group. These two bands have been regarded as characteristic peaks of polysaccharides (e.g., stachyose). The absorption peaks at approximately 3374 and 1647 cm^−1^ are typical of protein IR peaks (e.g., soy protein isolate). The appearance of a band at 1718 cm^−1^ due to the vibration of C=O groups provided evidence of the presence of phospholipids in the soy protein isolate and soy protein isolate-stachyose mixtures in the absence and presence of vitamin D_3_. The FTIR data reveal subtle perturbations in the majority of the frequencies due to lipid-protein interactions, which are likely induced by lipophilic bioactive compounds (e.g., vitamin D_3_). It should be noted here that a decrease in band intensity, as pointed out by an arrow in Figure 1, may be due to the incorporation of vitamin D_3_ onto the lipid bilayer.

### 3.4. XRD Study

**Figure 2:**
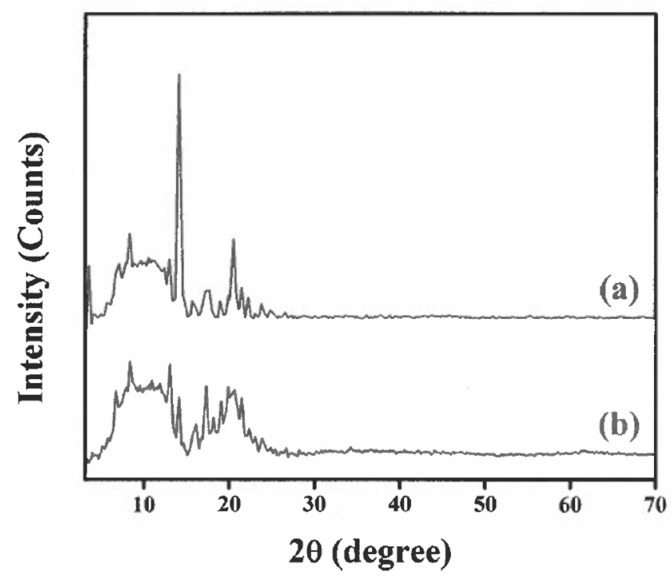
XRD of free vitamin D_3_ (**a**) and soy protein isolate-stachyose matrix loaded with vitamin D_3_ dispersed in DMSO (**b**) at different scattering angles (2θ).

The XRD measurements were performed to reveal the crystal nature of vitamin D_3_ and its transformation within the emulsion gel comprised of soy protein isolate and stachyose. Pure vitamin D_3_ showed crystal diffraction peaks ranging from 12 to 21 (Figure 2a). However, minor diffraction peaks were observed in the vitamin D_3_-loaded emulsion gel (Figure 2b). These later peaks were similar to the diffraction pattern of crystalline vitamin D_3_, but such peaks were weaker in intensity (Figure 2b). These changes clearly indicate a transformation of vitamin D_3_ from a crystalline state to an amorphous state in the vitamin D_3_-loaded emulsion gel. It is generally accepted that vitamin D_3_ in the amorphous state exhibits increased solubility and bioavailability. The amorphous state lacks the rigid crystal structure of the crystalline form, thereby allowing for faster gastric dissolution and being readily taken up by the cells in the body.

### 3.5. Physical Characterization

**Figure 3:**
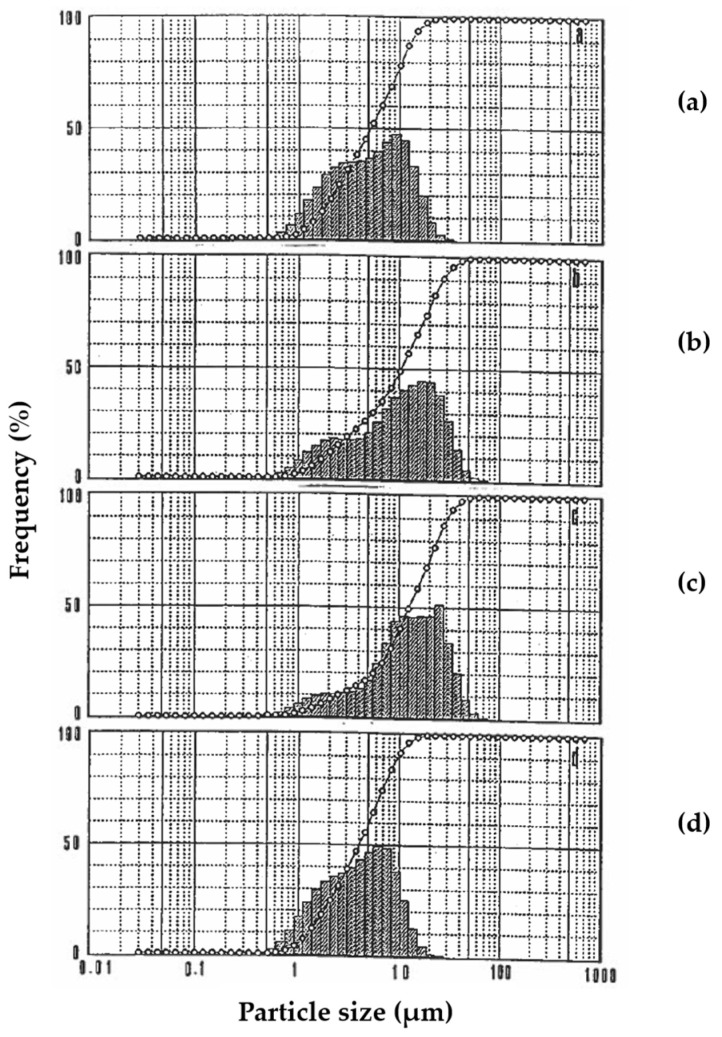
Particle size distribution of oil droplets in the soy protein isolate-stachyose emulsion gel loaded with vitamin D_3_ at 20 °C as a function of incubation time under simulated gastric fluid (SGF) at (**a**) 0 h, (**b**) 4 h, (**c**) 12 h, and (**d**) 24 h.

Figure 3 shows the particle size distribution of the oil droplets in the gelled emulsion to demonstrate that emulsions induced by dissolution of the gelled emulsion were stable at artificial gastric conditions. Thus, it is observed that the oil droplets of the gelled emulsions formed small droplet sizes with a mean diameter of approximately 5.34 μm and gave a homogeneous pattern at 0 h of gastric digestion (Figure 3a). The droplet size of the oil droplets in the emulsion gel became larger and showed more heterogeneous patterns at 4 h and 12 h of gastric digestion (Figure 3b and Figure 3c, respectively). On the other hand, the oil droplets of the emulsion gel formed smaller droplets at 24 h of gastric digestion (Figure 3d). These results indicate that the emulsifying activity and emulsifying stability of the soy protein isolate-stachyose gel emulsions loaded with vitamin D_3_ were affected by gastric digestion at different times of incubation. Moreover, a high absolute value of negative zeta potential was observed in the soy protein isolate-stachyose gel emulsions loaded with vitamin D_3_ at different times of gastric digestion, which provided enough electrostatic repulsion force to prevent droplet aggregation and improve dispersion stability, thereby resulting in a dense emulsion gel structure [30]. The zeta potential of these oil droplets ranged from −30.72 mV at 0 h to −39.84 mV at 24 h of gastric digestion.

### 3.6. NMR Spectroscopy

The water-binding property of soy protein isolate-stachyose emulsion gels is of great interest because the quantity of water associated with the polymer chains creates a unique environment that facilitates biological interactions. According to the hydration data measured by ^17^O NMR (Table 3), the addition of stachyose significantly (*p* < 0.05) improved the hydration properties of soy protein isolate in deuterated solutions at a pH/pD value of 2.4 (at 21 °C) in the presence of 0.4 M NaCl. However, at a pH/pD value of 7.6 (at 21 °C), the hydration properties of soy protein isolate differed (*p* < 0.05) only at a stachyose concentration of 0.3%. These ^17^O NMR results may be due to the shorter chain length and more hydroxyl groups of stachyose at lower pH/pD, which lead to better hydration. The number of water molecules interacting with the hydroxyl groups, through mechanisms including hydrogen bonding and chemical exchanges of protons, depends on the concentration of the stachyose polymer.

**Figure 4:**
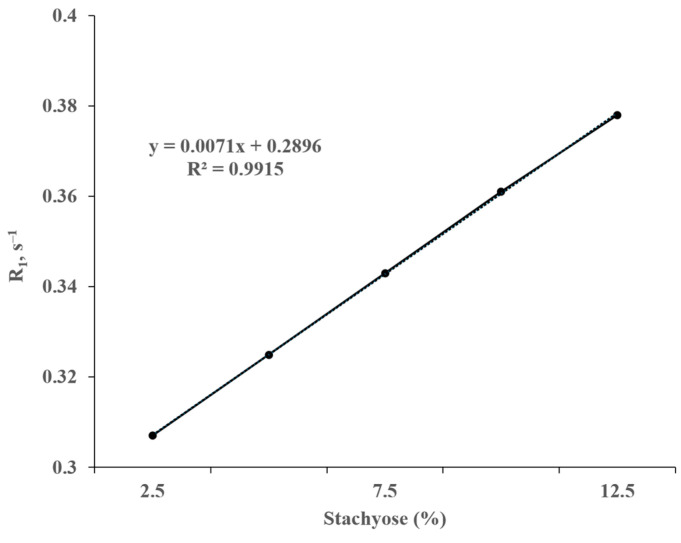
Correlation of the longitudinal relaxation rate (R_1_) of the water protons with increasing stachyose concentrations measured at 300 MHz, 21 °C, and pH/pD 6.5 in D_2_O. The straight line represents the least squares line of the best fit.

**Figure 5:**
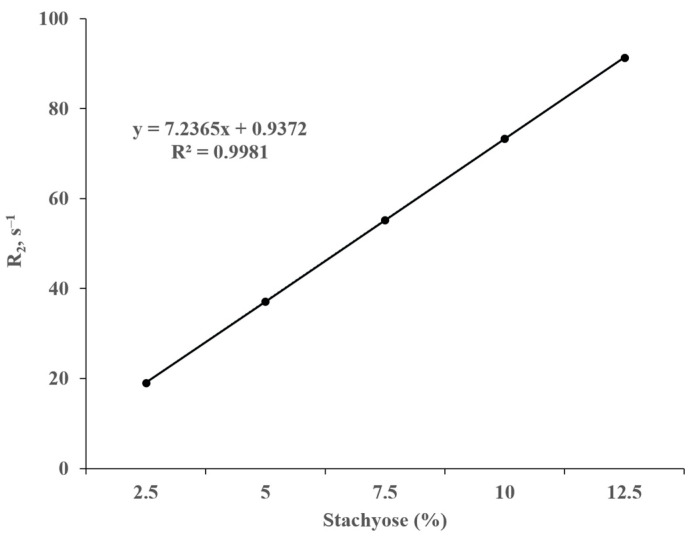
Correlation of the transverse relaxation rate (R_2_) of the water protons with increasing stachyose concentrations measured at 300 MHz, 21 °C, and pH/pD 6.5 in D_2_O. The straight line represents the least squares line of the best fit.

It was observed by high-field ^1^H NMR that the longitudinal and transverse relaxation rates (R_1_ and R_2_) of water protons decreased as the stachyose concentration increased at pH/pD 6.5 (Figure 4 and Figure 5, respectively). As shown in Figure 4 and Figure 5, both R_1_ and R_2_ exhibit a linear correlation with the concentration of stachyose. The slope of the line of best fit for the plot of R_2_ against stachyose concentration was almost 1000 times deeper than that of R_1_.

Translational relaxation of a nucleus is influenced by both the slow and fast motions of that nucleus, while the longitudinal relaxation is affected by fast motions only. R_2_ would be equal to R_1_ if the water molecules only exhibited fast motions. The fact that the value of R_2_ is greater than that of R_1_ for a given concentration of stachyose indicates that the water molecules also exhibit slow motions, which is consistent with the interaction of water protons with the hydroxyl groups of stachyose macromolecules. Moreover, Li et al. [13] noted that the addition of stachyose reduces the particle size of the soy protein isolate emulsion, thereby inducing the emulsion gel network to capture more water.

**Figure 6:**
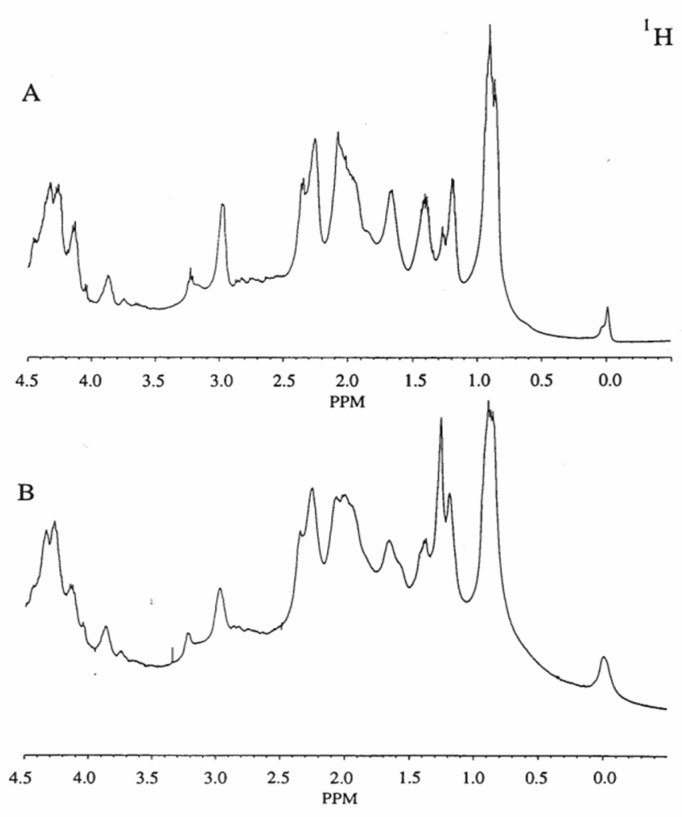
The upfield region of the ^1^H NMR spectra (400 MHz) of the soy protein isolate-stachyose gelled emulsion at concentrations of 2.5% *w*/*w* and 0.3% *w*/*w*, respectively, in D_2_O at 37 °C. (**A**) pH/pD at 2.4 and (**B**) pH/pD at 7.6. Each spectrum is the result of 500 scans; the applied exponential broadening is 0.5 Hz. Other experimental details are given in Section 2.

Our results indicate that water protons interact through chemical exchange processes with stachyose and do so differently according to pH/pD, as evidenced by ^17^O NMR (Table 1). These results provided a basis for understanding how water is bound by stachyose. The upfield region of the ^1^H NMR spectra (400 MHz) of the soy protein isolate-stachyose gelled emulsion sample at pH/pD 2.4 (Figure 6A) and 7.6 (Figure 6B) at 37 °C are almost identical. The absorbance changes were small, as no major rearrangement of protein conformation as a result of pH/pD could be detected.

### 3.7. In-Vitro Study

Vitamin D_3_ is susceptible to heat, light, and air (oxygen), which accelerates the degradation of vitamin D_3_ into less bioactive compounds, thereby reducing its health benefits [32]. In order to solve this issue, various stabilization strategies have been proposed in recent decades. However, each stabilization strategy has its own advantages and limitations. One stabilization approach is the use of soy protein isolate-stachyose emulsion gel as a vehicle for the delivery of vitamin D_3_ [13]. This is the first study, to our knowledge, reporting that vitamin D_3_ embedded within the soy protein-stachyose gel emulsion shows immune-boosting potential. The addition of stachyose to soy protein isolate had a major impact on their pharmacokinetic parameters, which have been ascribed to a better protection of the bioactive compound from gastrointestinal (GI) degradation and the controlled release of such bioactive compound [13].

**Figure 7:**
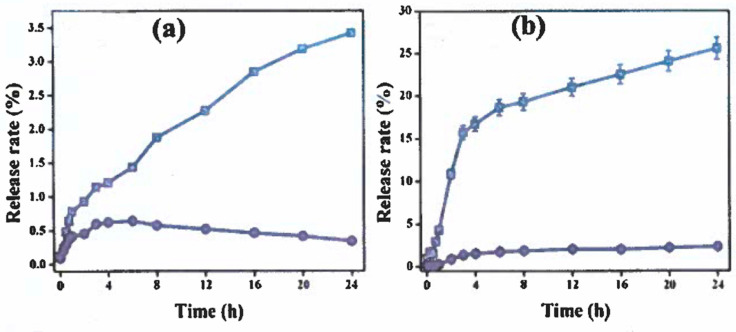
*In*-*vitro* release rate of free vitamin D_3_ (o) and vitamin D_3_ embedded within the soy protein isolate-stachyose delivery system (**☐**) under (**a**) simulated gastric fluid (SGF) and (**b**) simulated intestinal fluid (SIF).

The vitamin D_3_ profiles were performed between free vitamin D_3_ and vitamin D_3_ embedded within the soy protein isolate-stachyose delivery system for 24 h to verify the release characteristics. The in vitro simulated digestion experiments shown in Figure 7a clearly indicate that the soy protein isolate-stachyose delivery system slows down the release of vitamin D_3_ or delays its degradation in the stomach [32,33]. After a 24 h incubation period in SGF, vitamin D_3_ release rates were registered as 0.4% and 3.2% for free vitamin D_3_ and vitamin D_3_ embedded within the soy protein isolate-stachyose delivery system (Figure 7a). However, after digestion in SIF, the vitamin D_3_ release was significantly increased when compared with SGF, even though the vitamin D_3_ embedded within the soy protein-isolate-stachyose delivery system release rate (25.1%) was greater than the free vitamin D_3_ (2.6%), as shown in Figure 7b. In fact, the vitamin D_3_ release rates of the embedded vitamin D_3_ within the soy protein isolate-stachyose delivery system release rates in both SGF and SIF were greater than that of free vitamin D_3_. These results indicate that vitamin D_3_ embedded within the soy protein isolate-stachyose delivery system had a good sustainable release profile due to the incorporation of vitamin D_3_ into the soy protein isolate-stachyose delivery system and stabilized by the emulsion gel network, which is a suitable delivery system for vitamin D_3_ in good agreement with the earlier study [13].

### 3.8. In-Vivo Study

Vitamin D_3_ refers to two biologically inactive precursors: D_3_, also known as cholecalciferol, and D_2_, also known as ergocalciferol. The former produced in the skin on exposure to UVB radiation (290 to 320 nm) is known to be more bioactive. The latter is derived from plants and only enters the body via the diet. Both D_3_ and D_2_ precursors are hydrolyzed in the liver and kidneys to form 25-hydroxyvitamin D, the non-active storage form, and 1,25-dihydroxyvitamin D, the biologically active form that is controlled by the body.

In the present study, blood serum 25-hydroxyvitamin D_3_ [25-(OH)-D_3_] concentrations at the feeding period of 56 days were 23.71 ± 0.647 ng/mL for the basal diet, 27.24 ± 0.813 ng/mL for the basal diet supplemented with vitamin D_3_ dispersed in MCT oil, and 34.29 ± 1.550 ng/mL for the basal diet supplemented with vitamin D_3_ embedded within soy protein isolate-stachyose emulsion gel (Table 4). It is observed that the diet with vitamin D_3_ embedded within the emulsion gel (Diet 2) had significantly higher (*p* < 0.0001) blood serum 25-hydroxyvitamin D_3_ concentrations after 56 days of the feeding trial.

Heat stress is one of the main limiting factors of sustainable production in small ruminants, especially in hot and humid climates [34]. Heat stress occurs when animals are exposed to environmental conditions that exceed their ability to regulate body temperature effectively [34,35]. When temperatures exceed 30 °C, small ruminants begin to feel stressed, and above 35 °C, their ability to cool down through evaporative mechanisms becomes less effective. Body temperature and respiration rate signal when animals are under stress [34]. In our study, rectal temperature was evaluated through the study (Table 1). Heat stress acts on goats’ homeostasis. Due to the prevalence of infectious diseases in goats under heat stress, there has been interest in better immunity support as well as a demand for immune-enhancing functional materials. Vitamin D_3_ administration in the diet of goats could have a role in the enhancement of cell-mediated and humoral immune responses when goats are subjected to immune depression induced by heat stress. Thus, we sought to determine if OVA-immunized goats increased anti-OVA IgG responses between the two vitamin D_3_ dietary groups under heat stress.

When goats were fed the diet supplemented with vitamin D_3_ embedded within the soy protein isolate-stachyose emulsion gel for 56 days, a corresponding significant increase (*p* < 0.05) in anti-OVA IgG production was obtained. The humoral antibody response appears responsible for this activity. These studies show that specific IgG responses can be observed in cows and goats using OVA from chicken egg as previously indicated [17,29]. Our data indicate the role of vitamin D_3_ in the induction of IgG responses in healthy dairy goats under heat stress using OVA from chicken egg (Table 5). Particularly, the diet with vitamin D_3_ embedded within the emulsion gel (Diet 2) had significantly higher (*p* < 0.05) anti-OVA IgG concentrations after 14 days of the feeding trial.

Additionally, the exposure of the intestinal lining to the beneficial bacteria (e.g., *Bifidobacterium* spp.) may be able to induce a local immunomodulation [36]. Intestinal flora antigens prime the immunological tissues of the host so that a degree of nonspecific resistance toward infection is produced. Any protective effect, however, can be overcome by the entry of large numbers of pathogens [37].

The hydration results presented in Table 3 show that the soy protein isolate *per se* is much less “hydrated” (n_H_ = 0.00335 g of water/g of protein) than the soy protein isolate with added 0.3% stachyose (n_H_ = 0.00405 g of water/g of protein) at pH/pD 6.5 (at 21 °C) in deuterated phosphate-buffered saline (PBS) in the presence of 0.4 M NaCl. Interestingly, the addition of stachyose at 0.3% increases the degree of hydration of the soy protein isolate irrespective of the pH/pD in deuterated PBS (pH/pD 2.4, pH/pD 7.6) and ionic strength 0.4 M (NaCl), resulting in almost the same degree of hydration at both pHs/pDs (Table 3). Such hydration properties are expected to influence considerably the release behavior of vitamin D_3_ embedded within the soy protein isolate-stachyose emulsion gel. The hydration experiments also show that the biopolymers stabilizing the emulsion gel (e.g., soy protein isolate, stachyose) tolerate low pH/pD and ionic strength comparable to GI conditions.

## 4. Conclusions

The results suggest that high blood serum levels of 25-(OH)-D_3_ may be achieved by incorporating cholecalciferol (vitamin D_3_) in an emulsion gel comprised of the biopolymers soy protein isolate and stachyose prior to oral ingestion. The low dose of vitamin D_3_ present in the deliverable form of soy protein isolate-stachyose emulsion gel had a beneficial effect on the humoral immune response in OVA-immunized dairy goats under heat stress. The apparent high degree of hydration observed in the soy protein isolate-stachyose gel matrix at low pH and the prebiotic nature of stachyose are important in facilitating vitamin D_3_ interaction and modulating the composition of the microbiota, increasing in particular bacterial strains from the genus *Bifidobacterium*, which are health-promoting bacteria. Extended periods of studies in larger numbers of dairy goats under heat stress are needed to confirm these initial observations.

## Figures and Tables

**Table 1:** Rectal temperature of goats exposed to heat stress conditions from day 1 to 56.

Time (h)	Rectal Temperature (°C)		
	Control	Diet 1	Diet 2
0800	39.37	39.93	39.98
1200	39.82	39.67	39.89
1700	39.67	39.78	39.38
Average ± SD	39.62 ± 0.23	39.79 ± 0.13	39.75 ± 0.32

**Table 2:** Composition of basal diet used in feeding goats ^1^.

Item	Quantity
Dry matter (g/kg)	891
Crude protein (%)	18.1
Crude fat (%)	4.2
Fiber (%)	8.7
Total calcium (%)	0.9
Phosphate	0.6
Magnesium (%)	0.29
Manganese (mg/kg)	141
Vitamin A (IU/kg)	20,296
Vitamin D (IU/kg)	4428
Vitamin E (IU/kg)	68
Selenium (mg/kg)	0.8
NDF (%)	24.1
ADF (%)	11.1
NE_L_ (Mcal kg^−1^)	1.68

NDF, neutral detergent fiber; ADF, acid detergent fiber; NE_L_, net energy of lactation. ^1^ From Núñez de González et al., 2020 [27].

**Table 3:** Hydration n_H_^1^ estimates (g of water/g of protein) of soy protein isolate with added stachyose ^2^.

Stachyose (%)		pH/pD ^1^	
	2.4	6.5	7.6
0	0.00458 ± 0.000349 ^c^	0.00335 ± 0.000306 ^c^	0.00687 ± 0.000090 ^b^
0.1	0.00691 ± 0.000037 ^b^	0.00349 ± 0.000112 ^c^	0.00730 ± 0.000642 ^ab^
0.2	0.00721 ± 0.000408 ^b^	0.00382 ± 0.000066 ^c^	0.00746 ± 0.000277 ^ab^
0.3	0.00829 ± 0.000197 ^b^	0.00405 ± 0.000160 ^c^	0.00861 ± 0.000160 ^a^

^1^ From ^17^O NMR data (at 21 ± 1 °C) and at pH/pD 2.4, 6.5, and 7.6 in 0.4 M NaCl according to a two-state isotropic model [31]. ^2^ Data are presented as means ± SE. ^a–c^ Means in the same column with different superscripts are different (*p* < 0.05).

**Table 4:** Serum concentrations (ng/mL) of 25-hydroxyvitamin D_3_ [25-(OH)-D_3_] in dairy goats fed a basal diet and experimental diets ^1^ at baseline (0 d) and at feeding period (56 d).

Diet ^2^	Baseline (0 d) ^2^	Feeding Period (56 d)	*p*-Value
Control ^1^	21.71 ± 0.644	23.71 ± 0.647	0.229
Diet 1	21.41 ± 1.470	27.24 ± 0.813	0.001
Diet 2	21.75 ± 0.462	34.29 ± 1.550	<0.0001

^1^ Dairy goats in midlactation under heat stress fed a basal diet (control), vitamin D_3_ supplementation dispersed in MCT oil (Diet 1), and vitamin D_3_ embedded within the emulsion gel (Diet 2). ^2^ Data are presented as means ± SE; *n* = 18.

**Table 5:** Anti-OVA IgG concentrations (mg/mL) in dairy goats under heat stress by interactions of experimental diet × sampling time ^1^.

Sampling Time (Days)	Experimental Diets ^2^		
	Control (*n* = 6)	Diet 1 (*n* = 6)	Diet 2 (*n* = 6)
0 ^1^	1.90 ± 0.213 ^c C^	1.87 ± 0.184 ^c C^	1.93 ± 0.080 ^c D^
14	1.93 ± 0.180 ^c C^	1.90 ± 0.103 ^c C^	1.98 ± 0.217 ^c D^
28	6.20 ± 1.168 ^c A^	11.67 ± 2.106 ^b A^	17.57 ± 2.635 ^a C^
42	3.22 ± 0.796 ^c B^	13.83 ± 1.376 ^b A^	23.83 ± 1.487 ^a B^
56	2.57 ± 0.403 ^c C^	8.37 ± 2.345 ^b B^	30.08 ± 1.101 ^a A^

^1^ Dairy goats in midlactation under heat stress fed a basal diet (control), vitamin D_3_ supplementation dispersed in MCT oil (Diet 1), and vitamin D_3_ embedded within the emulsion gel (Diet 2). ^2^ Data are presented as means ± SE; *n* = 18. ^a–c^ Means in the same row within each sampling time with different lowercase superscripts differ (*p* ˂ 0.05). ^A–D^ Means in the same column within each experimental diet with different uppercase superscripts differ (*p* ˂ 0.05).

## Data Availability

The data presented in this study are available on request from the corresponding author.
